# Scaling-up production of cost-effective and eco-friendly bio-fertilizer and its application on Barley green fodder via IoT hydroponic system

**DOI:** 10.1186/s43141-021-00196-1

**Published:** 2021-06-28

**Authors:** Mustafa Elsayed Abd Elsallam, Shahira Hussainy EL-Moslamy, Ahmed Abd El-Al, Hoda Farouk Zahran

**Affiliations:** 1 Agricultural Engineering Department, Faculty of Agriculture, EL-Shatby, Alexandria University, Alexandria, Egypt; 2 Bioprocess Development Department, Genetic Engineering and Biotechnology Research Institute (GEBRI), City of Scientific Research and Technological Applications (SRTA-City), New Borg Al-Arab City, Alexandria 21934 Egypt; 3 Pollution Management Department, Environment and Natural Materials Research Institute, City of Scientific Research and Technological Applications (SRTA-City), New Borg Al-Arab City, Alexandria 21934 Egypt

**Keywords:** Bio-fertilizer, Barley, Hydroponic system, Endophytic *T. harzianum*, Fed-batch fermentation, IoT

## Abstract

**Background:**

Plant-associated microbes (endophytes) have a significant relationship to enhance plant growth and crop productivity by producing proficient bioactive metabolites. Since endophytes promoted plant growth either directly by releasing active metabolites such as phytohormones or indirectly by suppressing the growth of phytopathogens, so, in this work, biomass yield of local endophytic *Trichoderma harzianum* was maximized at shake-flask scale and scaled up via 7-L Bioflo310 fermenter using continuous exponential fed-batch fermentation mode. Subsequently, the effect of these cells as bio-fertilizer was assessed using two-barley grain genotypes (Russian and Egyptian seeds) via an intelligent hydroponic system based on Internet of Things (IoT).

**Results:**

To reduce the cost of a biomass production line, agro-waste media containing potato, onion, garlic, pea, and cabbage peels were chosen as the culturing medium. The pea peel medium was found to be the best producer of biomass (2.2 g/L). The cultivation factors were evaluated to improve this biomass yield. The results showed that the maximum biomass production (4.9 g/L) was reported by adjusting the medium pH at 5.0 that inoculated with 10% of spore suspension, then incubated at 30°C, and 200 rpm. Then, this biomass yield was scaled up kinetically (505.4 g/L) by using exponential fed-batch fermentation mode via a 7-L bioreactor. The stimulation impacts of this endophytic *T. harzianum* on the growth of different barley genotypes (Russian and Egyptian seeds) were determined using a controlled hydroponic chamber. The total chlorophyll, carotenoid, and carbohydrate amounts in treated Russian showed the proficient stimulation percentage (81.05, 80, 40.8%) compared to the Egyptian barley groups (76.39, 73.5, 25.9%) respectively. Also, the maximum carbohydrate content (83.95 ± 1.7%) was recorded in the case of Russian barley.

**Conclusion:**

Via this work, the optimal combination conditions for the cost-effective biomass production of endophytic *T. harzianum* were designed industrially via a fed-batch fermentation system using the cheapest culturing medium. Furthermore, by applying this promising bio-fertilizer, the total cost of barley production via an IoT hydroponic growing system was reduced. Besides, these animal diets (sprouted barley) could be produced in 3 cycles per month.

## Background

Plant-associated microbes that live asymptomatically are called endophytes. These microorganisms play a key role in enhancing plant growth and crop productivity by generating high-quality bioactive metabolites [1–3]. Therefore, these endophytes were applied as bio-fertilizers, bio-pesticides, or bio-remediators [3–7]. To increase crop production by using cheaper alternative solutions, efficient endophytes have prospected recently [8]. Several studies have studied the impacts of microbial bio-fertilizers on crop productivity and soil fertility enhancement through several pathways, such as nitrogen fixation, solubilization of phosphates, and critical nutrient accumulation. Several microorganisms, such as *Bacillus* sp., *Pseudomonas* sp., and *Trichoderma* spp., have been identified and evaluated to enhance various crop yields and to suppress phytopathogens using open field conditions [8–10]. Previously, there are different rhizosphere *Trichoderma* spp. such as *T. harzianum* and *T. viride* that were reported as bio-fungicides to eliminate disease-causing fungal pathogens. In the last few decades, many natural products (*Trichoderma* extracts) have become commercially available as bio-fungicides for controlling various phytopathogens such as *Botrytis* and *Fusarium* [9, 11–13]. There are several challenges to commercially scaling up the production of microbial biomass such as selecting low-cost, easily, and available nutrient-rich materials [8]. Additionally, the tested microbial cells must survive multiple processing stages without destroying their quality and quantity [14]. Therefore, there are low-cost materials such as agro-wastes that have been utilized as carbon and nitrogen sources for culturing microbial cells to reduce production line costs [14–17]. For the commercial scale-up approach, two distinct fermentation systems called solid-state and submerged fermentation were applied accordingly to the sensitivity of microbial cells. Through solid-state fermentation, the microbial cells are cultivated using non-soluble organic solid substrates as nutrient sources [16, 17]. This fermentation system requires lengthy fermentation periods, which increase the cost of the manufacturing line. A majority of enterprises have implemented a submerged fermentation method because some microbial cells were cultured using soluble carbon and nitrogen sources that were mixed with inducer supplementations. In this system, all microbial cultivation and condition parameters were managed and monitored automatically using bioreactor software [18]. Many fermentation modes were used to scale up production microbial cell density such as batch and fed-batch modes. When the cultivation process was completed without adding any extra supplemented nutrition, this mode is called batch fermentation mode. The fed-batch fermentation mode was implemented to increase microbial biomass densities by feeding extra substrates and other supplements after a certain period of incubation time [15]. One of the most significant cereal grains that are grown in diverse weather conditions worldwide is barley (*Hordeum vulgare* L) that belonged to the Poaceae family [19]. Barley ranks fourth in the world’s highest production of dry matter, following maize, wheat, and rice [20]. Barley crop is a significant source of food for a vast number of people and is also used as animal feed, industrial raw materials for some food, and drinkables. Several biotic and abiotic conditions affected badly the yield of crops; thus, various agricultural conditions need to be controlled for seed germination such as water level, oxygen, moisture, temperature, and light [21]. The ideal climate for the planting process cannot be ideally accomplished, and available lands for farming are decreasing. Lately, IoT was established in commercial production, utilities, and financial planning for healthcare applications [22–24]. So, it should come as no surprise that IoT would strongly be applied in agriculture as well. So, farmers need to be clever in the management of different crops by using smart agricultural systems to maximize crop productivity [25]. An automated IoT system has been designed using very sensitive sensors to monitor various agricultural conditions, such as humidity, temperature, pH, and light/dark cycles [26]. Therefore, this work aims to develop a cost-effective production line for scaling-up endophytic *T. harzianum* biomass weight. Then, the impacts of endophytic *T. harzianum* as bio-fertilizer are assessed using two-barley grain genotypes (Russian and Egyptian seeds) via an intelligent hydroponic system based on IoT.

## Methods

### Cultivation of endophytic *Trichoderma harzianum*

In this study, the bioactive metabolites were extracted from endophytic *Trichoderma harzianum* SYA.F4 (Accession number KX084391, https://www.ncbi.nlm.nih.gov/nuccore/1036392272) that was kindly provided by Dr. EL-Moslamy. Since different agro-wastes such as potato, onion, garlic, pea, and cabbage peels were tested for the cultivation of endophytic *T. harzianum* via solid-state and submerged fermentation strategies [27], in the case of solid-state fermentation, 40 g of the cutting peels was immersed separately into 4% glucose using tap water into a 250-mL conical flask. But in the case of submerged fermentation mode, 400 g of cutting peels mixed individually with 40 g of glucose and 1 L tap water was boiled for 15 min then mashed and filtered. The collected supernatants were completed to 1000 mL by adding tap water. These supernatants were divided into 100 mL for each 250-mL conical flasks. All of these solid and liquid flasks were sterilized at 121°C for 15 min then inoculated with five blocks (5 mm) of a 72-h-old culture of endophytic *T. harzianum*. The flasks that contained the solid agro-wastes were incubated statically at 28 ± 2.3°C for 3 weeks, but others were incubated at 30 ± 0.5°C in an orbital shaker of 150 rpm speed for 72 h. Subsequently, the highest fungal biomass yielding flask was determined statistically using MINITAB® Release 14.20.

### Optimization of the *T. harzianum* culturing conditions

Many culturing parameters were affected by the final biomass yielding such as pH, inoculum size, temperature, and agitation. So in work, the tested submerged cultures were prepared by adjusting the pH at different values such as 4, 5, 6, or 7. After sterilization, these flasks were inoculated using different inoculum sizes (1, 5, 10, and 15%) and incubated at 20, 27, 30, or 37 °C under different agitation speeds (0, 100, 200, 300, or 400 rpm). Finally, the collected fungal biomass was dried using an oven at 60°C for 24 h and the mean of dry biomass was calculated statistically.

### Scaling-up production of *T. harzianum*

In these experiments, upstream and downstream steps for scaling-up production were performed using fed-batch fermentation mode via 7-L Bioflo310 fermenter (New Brunswick Scientific Co., USA). This calibrated bioreactor was sterilized at 121°C for 20 min. Subsequently, the used temperature and pH were adjusted at 30°C and 5 respectively. The pre-inoculum (400 mL) was prepared by inoculating the prepared spore suspension (1.5 × 10^8^sp/mL) and cultivation at 30°C and 200 rpm for 72 h. At that moment, this pre-culture was inoculated into a prepared bioreactor (4600 mL) under aseptic conditions. During the cultivation process, the dissolved oxygen concentration was maintained above 30% using airflow (vvm) with agitation speeds (rpm) that were controlled automatically via Bioflo 310 software. The fungal growth curve was determined and the feeding step started at the late log phase by adding sterilized glucose solution (400 g/L) exponentially since the feeding rate started at 0.1 g/h. The maximum biomass dry weight (X_max_) was determined statistically using withdrawn samples. Additionally, the behavior of the tested fungal cells was described kinetically by calculating the yield coefficient (Y_X/S_) and the maximum specific growth rate (μ_max_) via Monod equations.

### Hydroponic system for treated grain sprouting

**Fig. 1:**
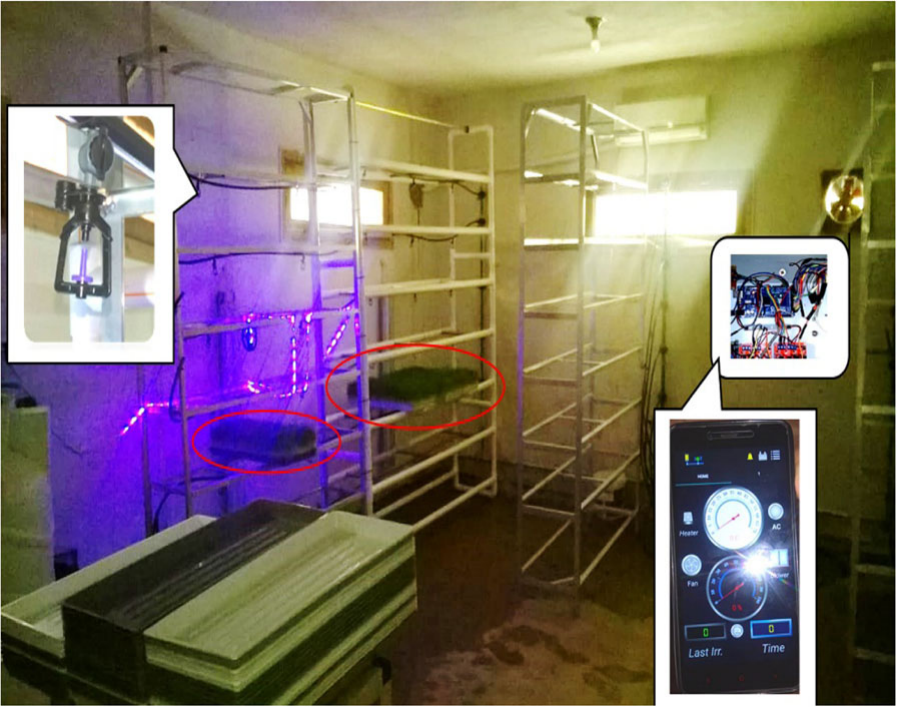
Intelligent hydroponic chamber based on Internet of Things (IoT)

In this work, a growing system was planned by using a fully controlled hydroponic chamber as shown in Fig. 1 with the next specifications: the sizes of 3.0 × 5.0 × 7.0 m consisted of 6 stands with 7 levels; each level accommodates 6 polyethylene trays (70 × 30cm) equipped with automatic sprayer irrigation (36 L/h). For barley grain sprouting, the used temperature and humidity were controlled using air circulation from 18 to 25°C and 70–85% respectively. Additionally, the light system consisted of fluorescent lighting tubes arranged in both vertical and horizontal positions and provided 1000–1500 microwatt/cm^2^ during 15 h of daily light. In these experiments, Russian and Egyptian seeds were selected to test, since these barley genotypes are considered agricultural and industrially important crops. Firstly, a set of these seeds were sterilized and immersed in spore suspension of *T. harzianum* (1 × 10^8^/mL), but another set of seeds were soaked in tap water for 24 h. Subsequently, these seeds were spread on trays (1.25 kg/tray); then, distributed randomly with three replicates. Secondly, the barley growth conditions were controlled and monitored automatically for 2 weeks. During that, many parameters such as fresh weight, dry weight, shoot length, root length, and fresh weight of trays were determined statistically. Then, crude fats, crude protein, total chlorophyll content, carbohydrate content, carotenoid content, fibers, moisture, and ash were estimated according to standard methods [28–32]. These analyses were carried out for the comparisons using a simple analysis of variance via a one-way ANOVA test at (*P*-value < 0.05) level of significance using MINITAB® Release 14.20 (2005 USA).

## Results

### Cultivation of endophytic *T. harzianum* using agro-wastes

**Fig. 2:**
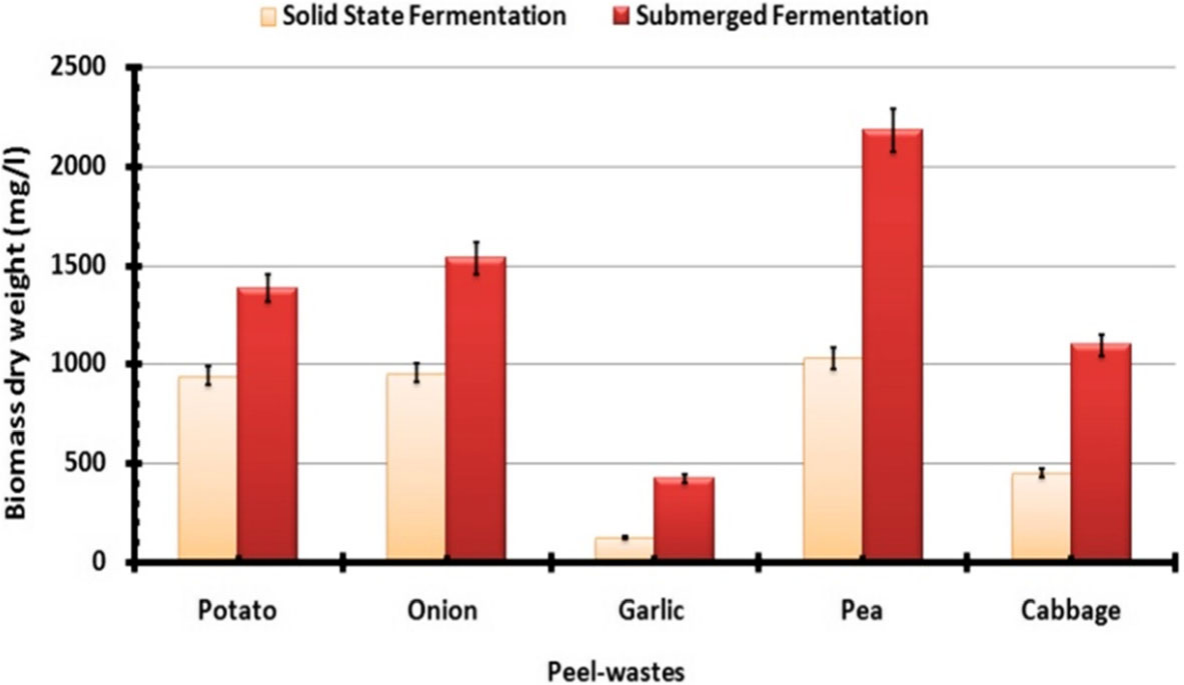
Effects of different waste peels on the produced biomass dry weight of endophytic *T. harzianum* cultivated by using submerged and solid-state fermentation

In this work, cost-effective large-scale mass production can be achieved by using agro-wastes (cheap and easily available) as carbon and nitrogen sources for fungal cultivation. The results obtained for endophytic *T. harzianum* mass production by using different agro-wastes such as potato, onion, garlic, pea, and cabbage peels via solid-state and submerged fermentation modes are shown in Fig. 2. These results show significantly (*P* ≤ 0.05) highest mass production was recorded by using pea peel (2.2 g/L) followed by onion peel (1.5 g/L) and potato peel (1.4 g/L) via submerged fermentation mode.

### Optimization of culture conditions for endophytic *T. harzianum*

**Fig. 3:**
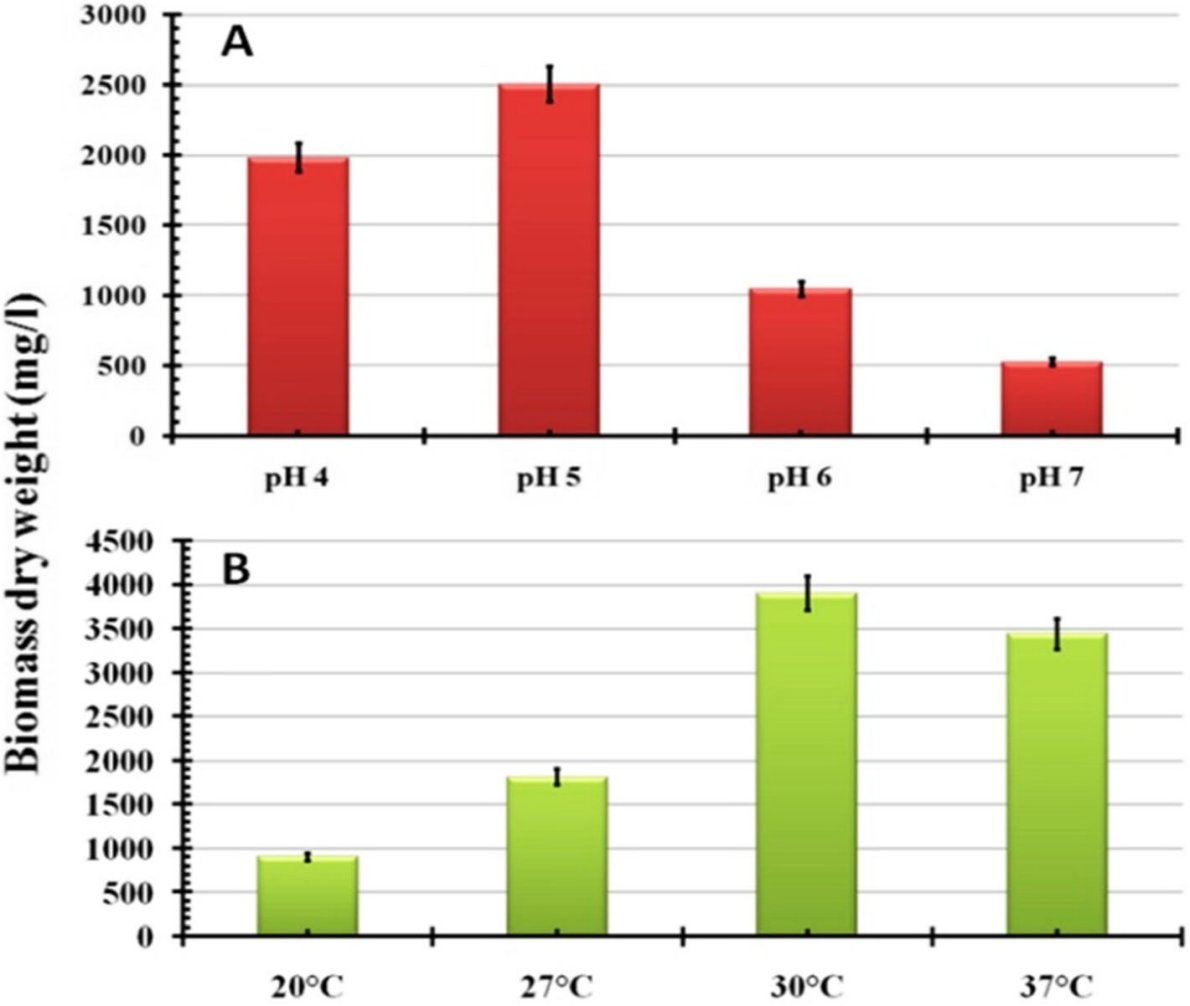
Detection of the effect of culturing parameters on biomass dry weight of endophytic *T. harzianum*, **a** pH, and **b** incubation temperature

**Fig. 4:**
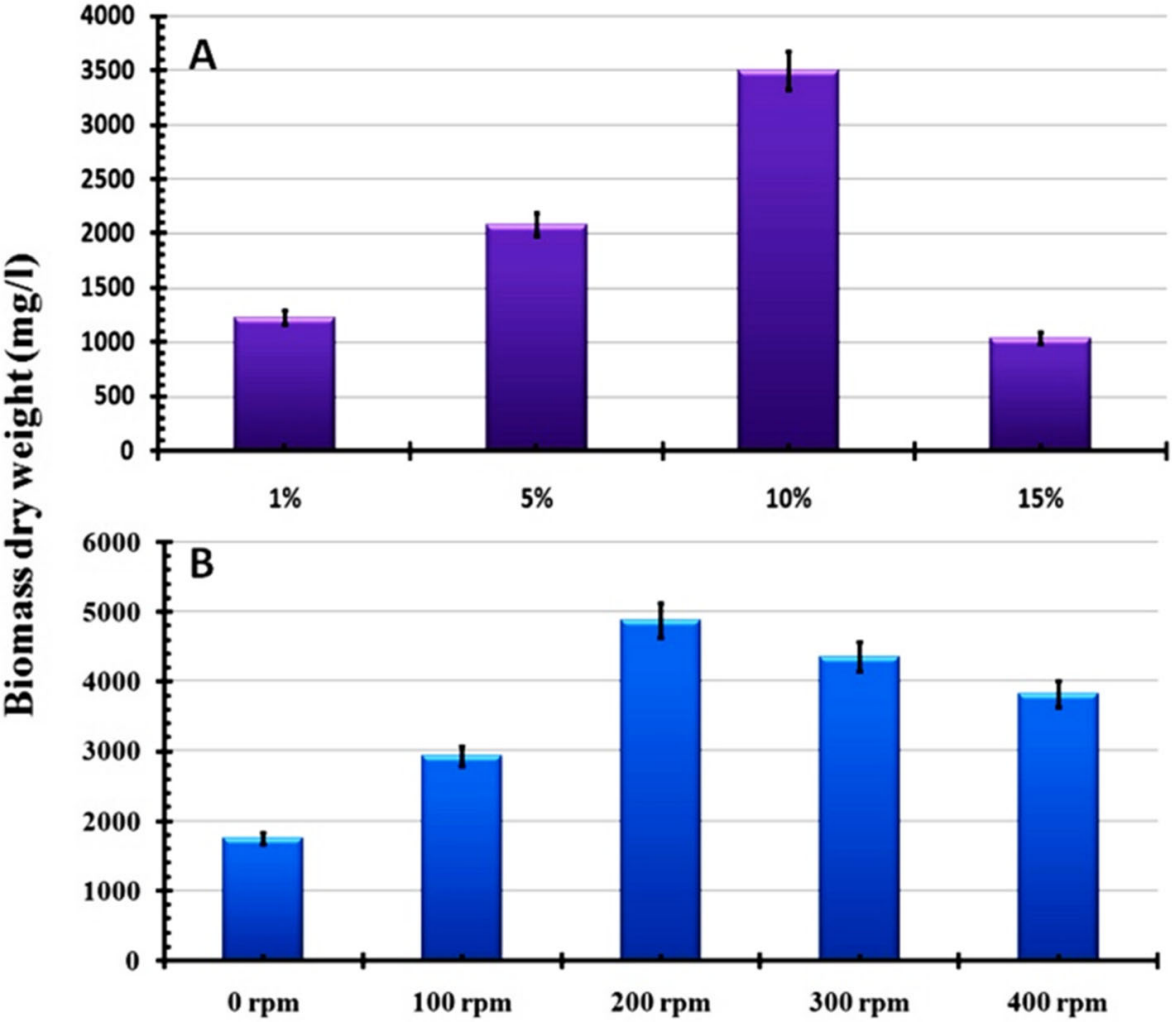
Evaluation of the effect of culturing parameters on biomass dry weight of endophytic *T. harzianum*, **a** inoculums size, and **b** agitation speed

This section was aimed to optimize the culturing conditions such as pH, inoculum size, incubation temperature, and agitation speeds for maximizing mass production of endophytic *T. harzianum.* Overall, the obtained results showed that the maximum mass production (4.9 g/L) in all studied culture conditions was recorded at pH 5.0 (Fig. 3a), 30°C (Fig. 3b), 10% of inoculum size (Fig. 4a), and 200 rpm (Fig. 4b).

### Implementation strategies for cultivation of endophytic *T. harzianum* via bioreactor

**Fig. 5:**
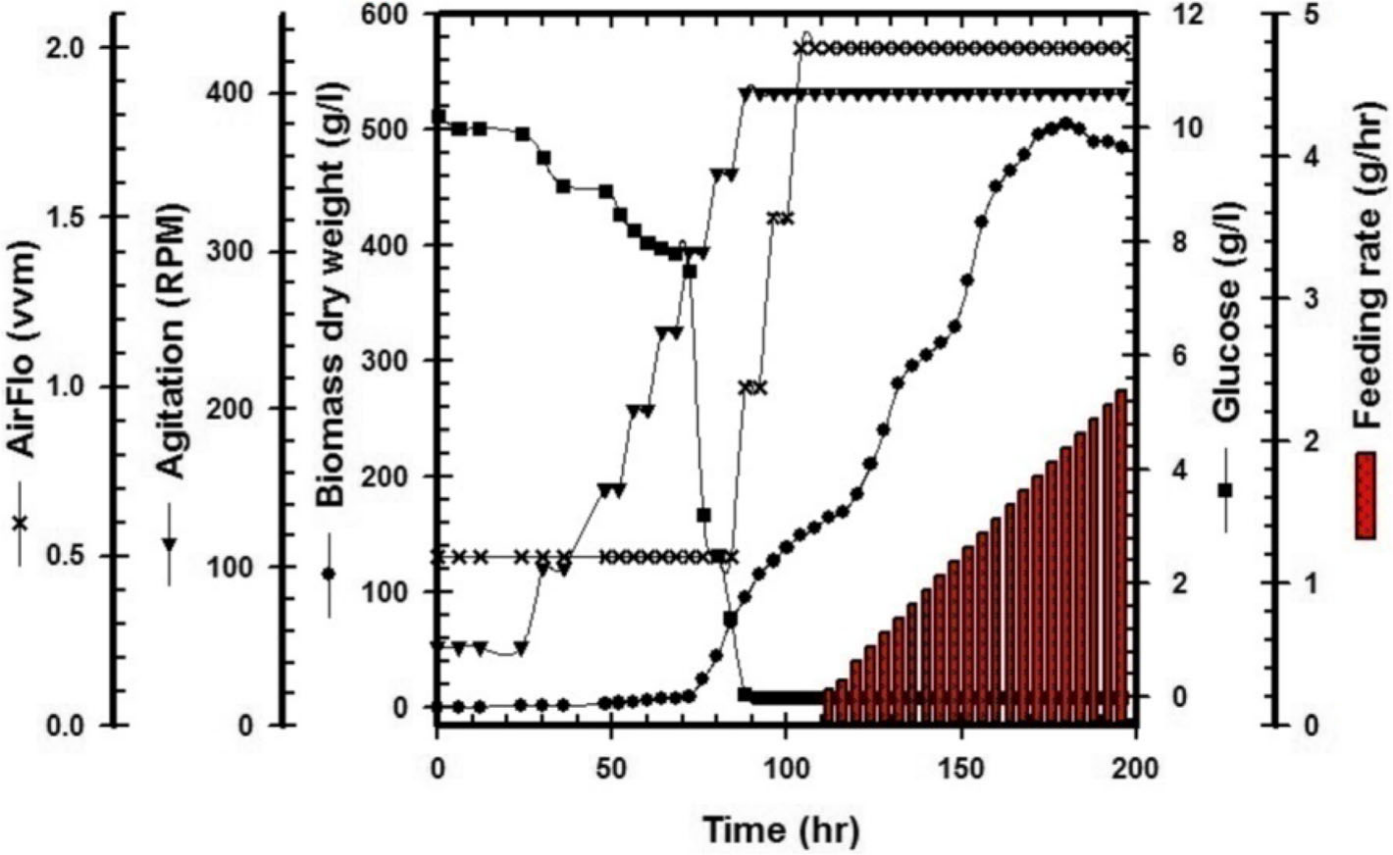
Time course of biomass dry weight (g/L), glucose consumption (g/L), agitation rate (RPM), airflow (vvm), and feeding rate (g/h) based on batch and fed-batch cultivation of endophytic *T. harzianum* using a 7-L Bioflo310 bioreactor. The feeding commenced at 80 h after batch cultivation and continued exponentially up to 196 h

In these experiments, the growth curve of endophytic *T. harzianum* was studied to detect perfect conditions for transposing its cultivation from shake flask to the large production scale. Since the batch and exponential fed-batch fermentation modes were applied and the behavior of the fungal cells was described kinetically, the yield coefficient was calculated by using Eq. (1) and the specific growth rate μ (h^−1^) is independent of nutrient concentration as shown in Eq. (2) that was used to calculate the maximum the specific cell growth rate (Eq. 3). Finally, the feeding rate was calculated by using Eq. (4) that was calculated by fixing the value of a specific growth rate. During the batch cultivation system, glucose was completely consumed after 72 h, and X_max_ was recorded as 84.6 g/L at 75 h; after feeding mode, the X_max_ was recorded as 505.4 g/L at 180 h as shown in Fig. 5.

1 $$ {\boldsymbol{Y}}_{\raisebox{1ex}{$\boldsymbol{X}$}\!\left/ \!\raisebox{-1ex}{$\boldsymbol{S}$}\right.}=\frac{\Delta  \boldsymbol{X}}{\Delta  \boldsymbol{S}}=\frac{\boldsymbol{X}-{\mathbf{X}}_{\mathbf{0}}}{{\mathbf{S}}_{\mathbf{0}}-\boldsymbol{S}} $$

where ***Y***_***X/S***_ biomass yield on substrate, **X** cell concentration, **X**_**0**_ initial cell concentrations, **S** substrate, and **S**_**0**_ initial substrate concentration. **X** and **X**_**0**_ are biomass concentrations (g/L) at measuring time **t** and initial time **t**_**0**_ respectively. **S** and **S**_**0**_ are the consumed amounts of carbon source (g/L) at the same times mentioned previously.

2 $$ \frac{{\boldsymbol{d}}_{\boldsymbol{x}}}{{\boldsymbol{d}}_{\boldsymbol{t}}}=\boldsymbol{\mu} \boldsymbol{X},\kern1.25em \boldsymbol{X}={\boldsymbol{X}}_{\mathbf{0}}\kern0.75em \boldsymbol{at}\kern1em \boldsymbol{t}=\mathbf{0}\kern0.5em \to \mathbf{\ln}\left(\frac{\boldsymbol{X}}{{\boldsymbol{X}}_{\mathbf{0}}}\right)=\boldsymbol{\mu} \boldsymbol{t},\kern1em \boldsymbol{or}\kern1.25em \boldsymbol{X}={\boldsymbol{X}}_{\mathbf{0}}{\boldsymbol{e}}^{\boldsymbol{\mu} \boldsymbol{t}} $$

3 $$ \boldsymbol{\mu} =\frac{{\boldsymbol{\upmu}}_{\mathbf{max}}}{{\boldsymbol{K}}_{\boldsymbol{S}}}+\boldsymbol{S} $$

where **μ** specific cell growth rate (h^−1^), **μ**_**max**_ maximum specific cell growth rate (h^−1^), **S** substrate concentration (g/L), and **K**_**S**_ saturation constant (g/L) = **S** when **μ** = 1/2 **μ**_**max**_.

4 $$ \mathbf{X}\mathbf{V}={\mathbf{X}}_{\mathbf{0}}{\mathbf{V}}_{\mathbf{0}}{\mathbf{e}}^{\boldsymbol{\upmu} \mathbf{t}},\kern0.5em \frac{\mathbf{d}\left(\mathbf{SV}\right)}{\mathbf{d}\mathbf{t}}=\mathbf{0},\kern0.5em \frac{\mathbf{d}\left(\mathbf{SV}\right)}{\mathbf{d}\mathbf{t}}=\mathbf{F}{\mathbf{S}}_{\mathbf{0}}-\left(\frac{\boldsymbol{\upmu} \mathbf{X}\mathbf{V}}{{\mathbf{Y}}_{\raisebox{1ex}{$\mathbf{X}$}\!\left/ \!\raisebox{-1ex}{$\mathbf{S}$}\right.}}\right),\mathbf{F}=\frac{{\boldsymbol{\upmu} \mathbf{X}}_{\mathbf{0}}{\mathbf{V}}_{\mathbf{0}}{\mathbf{e}}^{\boldsymbol{\upmu} \mathbf{t}}}{{\mathbf{S}}_{\mathbf{0}}{\mathbf{Y}}_{\raisebox{1ex}{$\mathbf{X}$}\!\left/ \!\raisebox{-1ex}{$\mathbf{S}$}\right.}} $$

### Beneficial impacts of endophytic *T. harzianum* on barley sprouting using the hydroponic system

The impacts of endophytic *T. harzianum* on growing barley grains were tested by utilizing two-grain genotypes (Russian and Egyptian seeds) through a completely controlled hydroponic chamber. Then, various physiological parameters were analyzed statistically to recognize the possible improvement parts of the tested barley. All physiological parameters (root length, shoot length, and weight of utilized tray) were improved by applying the endophytic *Trichoderma harzianum* as a bio-fertilizer in both Russian and Egyptian grains and compared with the untreated grains (Table 1). Consequently, the fresh weight of the treated shoot system of the Russian barley grains was recorded as the most elevated weight (6.44 ± 0.64 g/plant) contrasted and the Egyptian grain (4.34 ± 0.64 g/plant). Moreover, the treated Russian plant (25.67 ± 1.61 cm) has the tallest green parts compared with the Egyptian plant (21.11 ± 1.46 cm). Additionally, the fresh weight of the tested root systems was likewise determined in the two cases. Their measurable outcomes detected that the treated Russian grains had the most noteworthy weight (3.57 ± 0.1 g/plant) than the Egyptian one (2.48 ± 0.15 g/plant) compared with the untreated plant. Also, the root length of the treated Russian grains (9.09 ± 0.26 cm) was expanded than the Egyptian grains (5.91 ± 0.13 cm). Subsequently, the fresh weights of the used trays were determined statistically. Notably, the treated Russian barley trays have shown up as the most noteworthy weight contrasting the Egyptian trays as shown in Table 1.

**Table 1:** Valuable effects of endophytic *T. harzianum* on physiological parameters of the barley plant that sprouted through the hydroponic system

Parameters	Russian barley		Egyptian barley	
	Treated ± SD	Control ± SD	Treated ± SD	Control ± SD
**Fresh weight of shoot (g/plant)**	6.44 ± 0.64	2.53 ± 0.17	4.34 ± 0.64	2.22 ± 0.27
**Dry weight of shoot (g/plant)**	0.66 ± 0.11	0.25 ± 0.02	0.56 ± 0.11	0.3 ± 0.043
**Fresh weight of root (g/plant)**	3.57 ± 0.1	1.1 ± 0.22	2.48 ± 0.15	1.09 ± 0.09
**Dry weight of root (g/plant)**	0.32 ± 0.0015	0.1 ± 0.008	0.29 ± 0.01	0.13 ± 0.002
**Root length (cm)**	9.09 ± 0.26	3.91 ± 0.24	5.91 ± 0.13	3.81 ± 0.24
**Plant height (cm)**	25.67 ± 1.61	14.89 ± 1.05	21.11 ± 1.46	14.92 ± 0.17
**Tray weight (kg)**	3.89 ± 0.19	1.6 ± 0.36	4.08 ± 0.12	1.95 ± 0.1

**Fig. 6:**
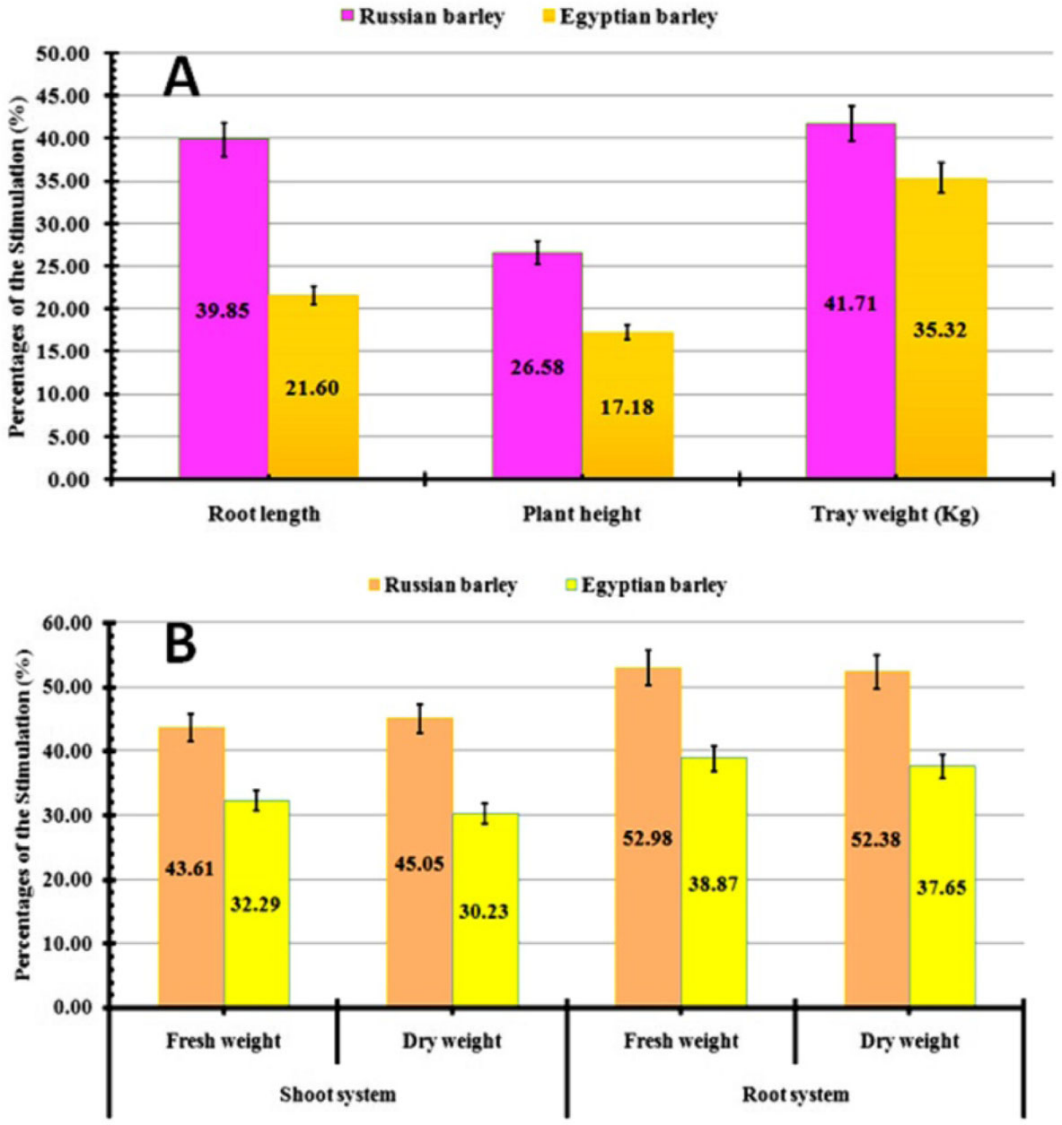
Physiological parameters of the sprouting barley plant that treated with endophytic *T. harzianum* through hydroponic system, (**A**): Root length, plant height, tray weight; (**B**): Shoot system, and root system weights

So, the tested endophytic *T. harzianum* has shown up its capacity to expand a root surface and collective length of roots, shoot, and leaf region compared with those recorded in controlled plants. Consequently, the stimulation percentages were calculated statistically using physiological growth parameters to finalize the proficient effects of tested endophytic *T. harzianum* in both cases as shown in Fig. 6. The highest stimulations were recorded at the Russian barley group as 41.71, 39.85, and 26.57% for tray weight, root length, and plant height respectively (Fig. 6a). Consequently, the root system has the highest stimulation effect (52.38%) compared with the shoot area (45.05%) as shown in Fig. 6b.

In this work, biochemical parameters were also statistically analyzed for the sprouting of checked barley varieties that were treated with endophytic *T. harzianum*. The tested parameters (fats, protein, fibers, moisture, ash, total chlorophyll, carbohydrates, carotenoid contents) and their effect are collected in Table 2. Generally, all parameters have been influenced positively by using endophytic *T. harzianum* relative to their controls. Comparing with the Egyptian group (68.75 ± 1.17%), the maximum carbohydrate content (83.95 ± 1.7%) was found in the Russian barley. In comparison, the proficient findings such as total chlorophyll (3.44 ± 0.12) and carotenoid contents (3.32 ± 0.43%) have been recorded in the Russian group relative to the control group (0.37 ± 0.2 and 0.36 ± 0.23% respectively). Higher fibers (40.35 ± 5.81%) and moisture contents (33.58 ± 2.65%) were recorded also in the Russian group than in the control group 15.51 ± 0.74 and 33.58 ± 2.65% respectively.

**Table 2:** Detection of the effect of the endophytic *T. harzianum* on biochemical metabolites of the sprouting barley plant using the hydroponic system

Parameters	Russian barley		Egyptian barley	
	Treated ± SD	Control ± SD	Treated ± SD	Control ± SD
**Crude fats (%)**	5.38 ± 0.182	1.59 ± 0.11	4.05 ± 0.99	1.46 ± 0.59
**Crude protein (%)**	16.81 ± 0.22	6.45 ± 1.85	19.85 ± 6.57	12.07 ± 1.99
**Total chlorophyll content (mg/g FW)**	3.44 ± 0.12	0.37 ± 0.2	2.56 ± 0.63	0.35 ± 0.11
**Carbohydrate content (%)**	83.95 ± 1.7	35.26 ± 5.14	68.75 ± 1.17	40.47 ± 1.81
**Carotenoid content (mg/g FW)**	3.32 ± 0.43	0.36 ± 0.23	2.51 ± 0.82	0.39 ± 0.08
**Fibers (%)**	40.35 ± 5.81	15.51 ± 0.74	32.53 ± 0.55	15.09 ± 0.96
**Moisture (%)**	33.58 ± 2.65	9.13 ± 1.01	26.96 ± 2.78	10.42 ± 0.73
**Ash (%)**	5.05 ± 1.09	1.67 ± 0.36	4.33 ± 1.25	1.75 ± 0.48

**Fig. 7:**
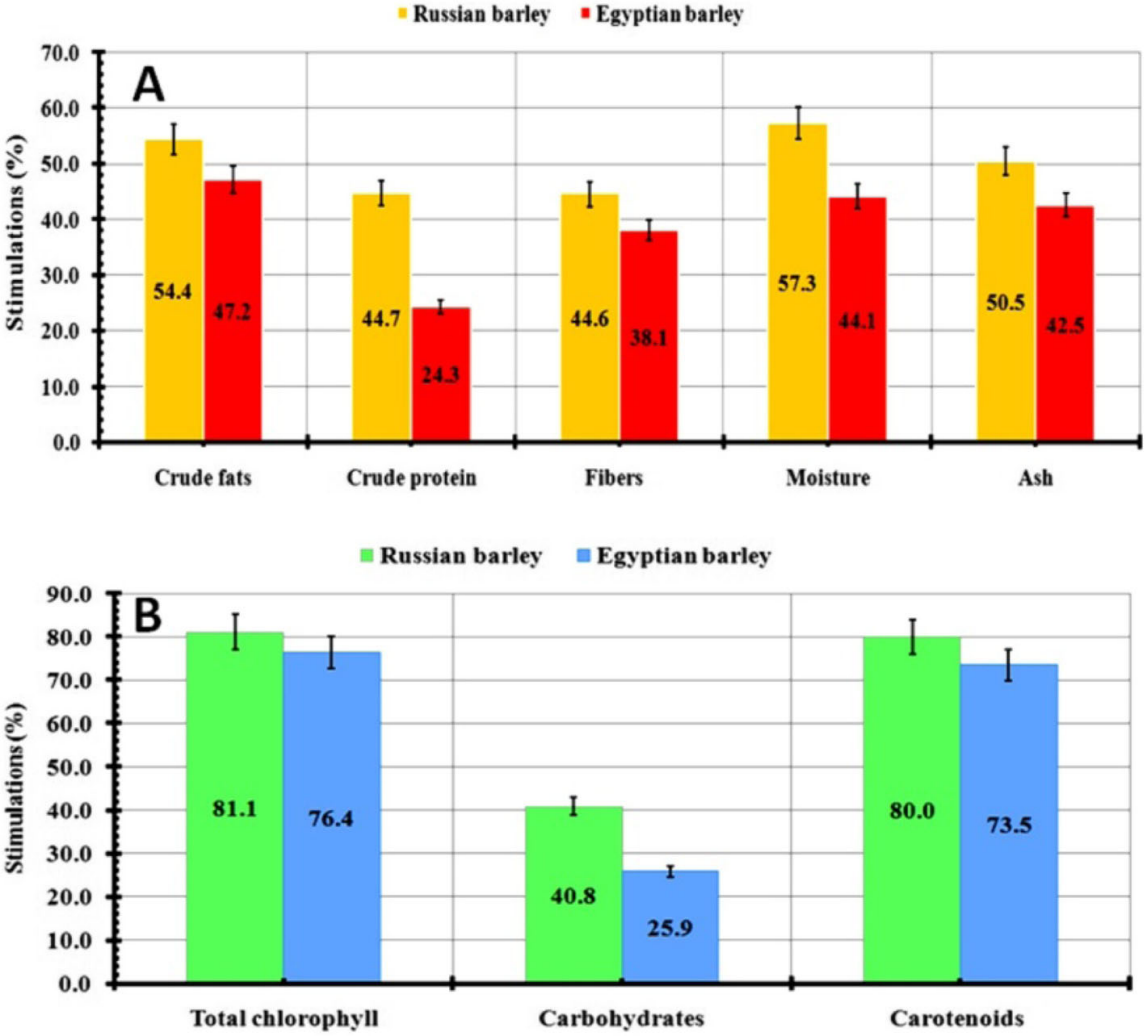
Biochemical metabolites of the sprouting barley plant that treated with endophytic *T .harzianum* through hydroponic system; (**A**): Crude fats, crude protein, fibers, moisture, ash; (**B**): Total chlorophyll, carbohydrates, carotenoids

**Fig. 8:**
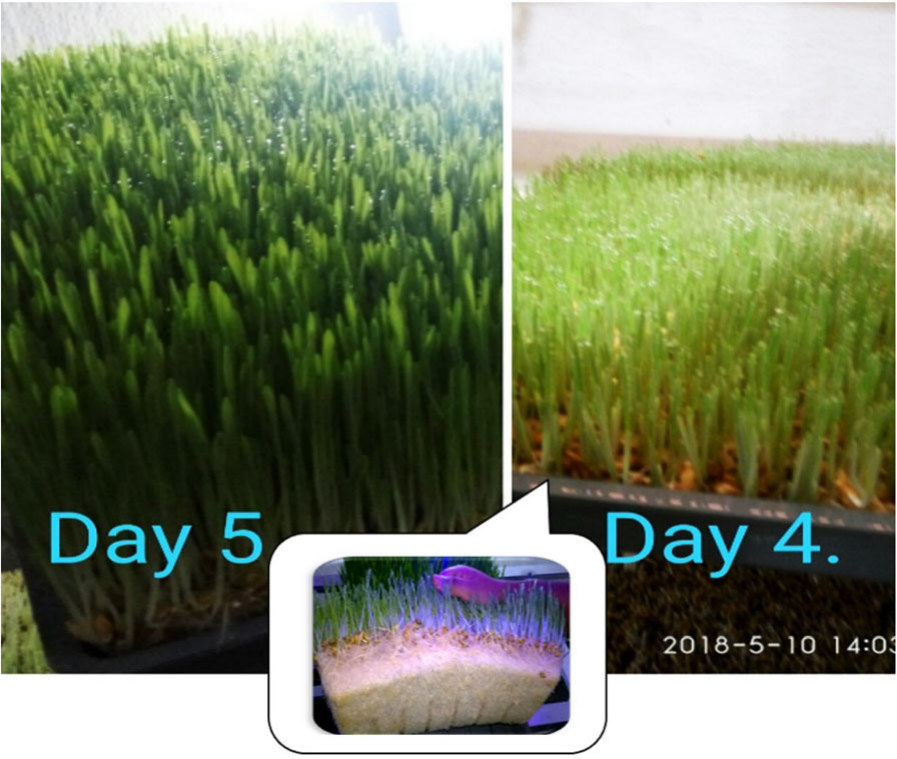
High productivity of Russian barely plant by using endophytic *T. harzianum* bio-fertilizer through the intelligent hydroponic system

Notably, this stimulation improved also the barley metabolism that was determined statistically by analyzing different biochemical parameters such as crude fats, crude protein, fiber contents, moisture, and ash (Fig. 7). In the case of Russian plants, the moisture content (57.3%) has higher stimulation effects than that in the Egyptian plant (44.1%). The data of crude fats, protein, and fibers in the Russian group were recorded as the perfect effect of the tested endophytic *T. harzianum* (54.4, 44.7, and 44.6% respectively) compared with the Egyptian one that calculated as 47.2, 24.3, and 38.1% respectively (Fig. 7a). Besides, the total chlorophyll, carotenoid, and carbohydrate amounts in the treated Russian group showed proficient stimulation percentages of 81.05, 80, and 40.8% compared to the Egyptian barley groups (76.39, 73.5, and 25.9%) respectively (Fig. 7b). It could be revealed from all the above findings that endophytic *T. harzianum* has a major stimulation effect on the growth parameters of the barley group studied, especially the Russian group. Therefore, the implementation of a hydroponic growing system using this cost-effective bio-fertilizer is a promising solution that reduces the overall cost of crop production by reducing the doses of the used growth regulators. In addition, by using this flexible and intensive hydroponic growing system, sprouted barley (animal diets) could be produced in 3 cycles/month (Fig. 8).

## Discussion

A *T. harzianum* has huge mixtures of primary and secondary bioactive metabolites, especially the endophytic group. So, it was considered a wonderful genus of fungi applied for promoting plant productivities and phytopathogens management [19]. For these extensive applications, the development of its scaling-up mass production and production line cost are major challenges of commercial production [33]. Previously, many agricultural wastes (vegetable and fruit wastes and different manures) were evaluated as cultivated media for mass production of different rhizosphere *Trichoderma* spp., such as *Trichoderma viride* and *Trichoderma harzianum*, through solid and submerged modes [27]. Through this work, mass production of endophytic *T. harzianum* (2.2 g/L) was achieved at a lab scale by using an inexpensive and widely available medium (wastage pea peel extract).

The variations in culturing conditions affected the fungal growth, sporulation, and germination especially via submerged fermentation mode using agro-wastes. Therefore, previous studies reported that the best agro-peels consumed for fungal mass production should possess the ability to produce the largest number of fungal propagates [34, 35]. So, the environmental conditions such as pH, incubation temperature, inoculum size, and agitation speed were optimized via our work. Then, the maximum mass production (4.9 g/L) was recorded by inoculated 10% of endophytic *T. harzianum* using wastage pea peel medium at pH 5.0 and incubated at 30°C under checking condition (200 rpm). Notably, by using these environmental conditions, there was approximately a twofold increase in the biomass production of tested endophytic *T. harzianum*.

Generally, any bioreactor consisted of a mechanical stirred vessel and control unit to manage a specific fermentation strategy. Then, different parameters should be controlled according to fermentation modes such as solid and submerged fermentation systems to increase microbial metabolites by applying growth kinetics equations [36]. At an industrial scale, the yield of mass production required ideal conditions using a relatively low-cost medium. Indeed, the key parameters such as pH, agitation, airflow, dissolved oxygen, temperature, and feeding pumps were affected by large-scale production of microbial mass production [16, 18]. Many studies have been reported to evaluate the cultivation parameters for *Trichoderma* sp., by using a submerged fermentation system to increase its bioactive metabolites as well as conidia [14–17]. To detect perfect conditions for transposing the cultivation of endophytic *T. harzianum* to the industrial scale, the batch and exponential fed-batch fermentation modes were applied in our work. Subsequently, the behavior of the fungal cells was described kinetically. Meanwhile, the 7-L Bioflo 310 bioreactor medium (pH, 5) was inoculated with spore suspension (1.5 × 10^8^sp/mL) and cultivated at 30°C. Additionally, the dissolved oxygen concentration was maintained above 30% using airflow (vvm) with agitation speeds (rpm) that were controlled automatically via bioreactor software. Subsequently, the feeding step started at the late log phase by adding sterilized glucose solution (400 g/L) exponentially since the feeding rate started at 0.1 g/h. Finally, X_max_ was recorded as 84.6 g/L at 75 h for the batch period, and after feeding mode, the X_max_ was recorded as 505.4 g/L at 180 h for the fed-batch period.

Plant growth-promoting microorganisms are considered significant eco-friendly solutions utilized for reducing agricultural issues. Endophytic fungi were reported as a proficient tool that was utilized broadly for improving the development of numerous crops, because they produced various bioactive metabolites that may boost soil fertility and plant immunity. As previously reported in [37–39], such stimulation can be due to either regulating minor phytopathogens and/or improving nutrient absorption (nutrient accessibility). Other studies have alleged that plant growth was stimulated by phytohormones, nutrients, auxins, and solubilizing minerals provided by *T. harzianum* [33, 37]. Beforehand, the rhizosphere *T. harzianum* was applied impeccably as a bio-fertilizer through numerous reports by utilizing different crops [7, 34, 37–39], for example, tomato, cucumber, pepper, lettuce and rocket, and barley and wheat.

Global warming and the availability of suitable soil are two factors that influence the yield of some crops. Aside from that, large quantities of fertilizers and pesticides are consumed, which harm the ecosystem when this traditional farming method is applied. To overcome these obstacles, hydroponics was used instead of the traditional method, by dissolving all minerals nutrients in water [40]. By optimizing agricultural conditions and nutrient doses using bio-fertilizers, different plants can grow faster and yields can be increased by using this hydroponic system [26, 41]. Furthermore, hydroponics is an effective method for conserving agricultural water, used pesticides, and fertilizers. So, there are many plants, including vegetables [25, 42, 43] such as onion, cucumbers, and lettuces, as well as different varieties of sorghum [44] that have been grown hydroponically with the manual management of human and/or computer. However, IoT is now being used to control hydroponic systems via mobile applications, eliminating the need for human or/and computer interaction [22, 23]. Because of the wireless sensors (such as humidity, water level, pH, and temperature) and monitoring tools that are connected to a web network, smart hydroponics may be remotely managed in real time to produce crops of desired quality and quantity [24, 43, 44]. Hydroponic green fodder is considered to be such an ideal way for planting seeds with high protein content and metabolic energy that is easily digestible by farm animals. Barley is an important raw resource for the feed market, and it is widely used for farm animal nutrition in the form of dry grains as well as sprouts. However, because hydroponically sprouted barley stimulates enzymes that convert the grain’s starch, protein, and lipids into simpler forms, it was preferred over dried seeds in many countries [43, 44].

Our analysis used two-grain genotypes (Russian and Egyptian seeds) in a fully controlled hydroponic chamber to test the effects of endophytic *T. harzianum* on growing barley grains. So, the tested endophytic *T. harzianum* has shown up its capacity to expand different plant regions compared with those recorded in controlled plants. Consequently, the stimulation percentages were calculated statistically to finalize the proficient effects in both cases. Consequently, the fresh weight of the treated shoot system of the Russian barley grains was recorded as the most elevated weight contrasted and the Egyptian grain. Moreover, the treated Russian plant has the tallest green parts compared with the Egyptian plant. As a result, using this cost-effective bio-fertilizer in a hydroponic growing system is a promising option for lowering the overall cost of crop production by lowering the doses of growth regulators used. Furthermore, sprouted barley (animal diets) could be grown in three cycles per month using this versatile and intensive hydroponic growing method.

## Conclusion

To reduce the cost of the industrial biomass production of endophytic *T. harzianum*, the optimal combination conditions were optimized via a fed-batch system using the cheapest culturing medium dependent on the pea peel. Besides, it is remarkably clear from this study that the application of endophytic *T. harzianum* as a cost-effective bio-fertilizer stimulates the production of barley growth, especially in the case of Russian seeds. So, by reducing the doses of used growth regulators, this promising bio-fertilizer will decrease the total cost of crop production. Thus, the animal diets (sprouted barley) could be produced in 3 cycles per month via this versatile and intense hydroponic growing system*.*

## Data Availability

All data generated and analyzed during this study are included in this manuscript.

## Abbreviations

IoT: Internet of Things
X_max_: Maximum biomass dry weight
Y_X/S_: Yield coefficient
μ: Specific cell growth rate
μ_max_: Maximum specific growth rate
vvm: Volume per volume per min
rpm: Round per min
X: Cell concentration
X_0_: Initial cell concentrations
S: Substrate
S_0_: Initial substrate concentration
X and X_0_: Biomass concentrations (g/L) at measuring time t and initial time t_0_
S and S_0_: The consumed amounts of carbon source (g/L)
K_S_: Saturation constant
F: Feeding rate
